# Small RNA *ASpks2* promotes *Mycobacterium tuberculosis* survival in macrophages *via* targeting *polyketide synthase 2*

**DOI:** 10.1016/j.jbc.2026.111449

**Published:** 2026-04-14

**Authors:** Qian Wu, Yifei Lu, Li Yuan, Ying Chen, Mingkun Yang, Feng Ge

**Affiliations:** 1 College of Life Science, Yangtze University, Jingzhou, China; 2 Key Laboratory of Breeding Biotechnology and Sustainable Aquaculture, Institute of Hydrobiology, Chinese Academy of Sciences, Wuhan, China

**Keywords:** *Mycobacterium tuberculosis*, polyketide synthase 2 (pks2), quantitative proteomics, small non-coding RNA (sRNA), virulence

## Abstract

*Mycobacterium tuberculosis* is the causative pathogen of human tuberculosis and a leading cause of death worldwide attributed to a single infectious agent. While small non-coding RNAs (sRNAs) have emerged as key regulators of bacterial pathogenicity, their specific roles and mechanisms in *M. tuberculosis* remain poorly understood. Here, we employed label-free quantitative proteomics, parallel reaction monitoring, and sRNA-seq analyses to identify proteomic differences between the virulent H37Rv and attenuated H37Ra strains. Bioinformatic analysis revealed significant enrichment of differentially expressed proteins involved in lipid metabolism and fatty acid biosynthesis, key pathways linked to *M. tuberculosis* virulence. We identified a novel sRNA, *ASpks2*, which was significantly downregulated in H37Rv. Functional validation demonstrated that *ASpks2* directly targets the *polyketide synthase 2* (*pks2*), modulating its expression to enhance *M. tuberculosis* survival in human macrophages THP-1 cells. By correlating the omics data with functional studies, this study identified a novel sRNA and its regulatory network in *M. tuberculosis*, which provides novel insight into the molecular pathogenesis of *M. tuberculosis* and may serve as a basis for the development of targeted therapies.

Tuberculosis (TB) persists as a formidable adversary to global health, with 10.6 million new cases reported in 2022 and an incidence rate of 133 per 100,000 individuals. TB claimed 1.3 million lives, making it the second leading cause of death from a single infectious pathogen, subordinate only to COVID-19, and approaching a twofold increment of HIV/AIDS (1). *Mycobacterium tuberculosis* primarily infects the lungs, and current TB control strategies face significant limitations. The *Bacillus* Calmette-Guérin (BCG) vaccine, while widely used, offers limited and inconsistent protection, particularly in adults (2). Standard treatment requires a six-month or longer regimen with multiple antibiotics, posing risks of hepatotoxicity and drug resistance. Alarmingly, only three new drugs targeting drug-resistant tuberculosis have been developed in the past 50 years, underscoring the urgent need for innovative research into the pathogenic mechanisms of *M. tuberculosis* (3).

The virulent *M. tuberculosis* H37Rv and its attenuated H37Ra strain serve as pivotal models for studying the molecular basis of *M. tuberculosis* pathogenicity. Genomic comparisons have identified variations in sequences such as IS6110 and the PE/PPE gene families as the primary drivers of genetic divergence (4, 5, 6). Jia *et al.* identified specific single-nucleotide polymorphisms (SNPs) associated with attenuated virulence, including S219L in PhoP, A219E in MazG, and the newly identified I228M in EspK (7). Transcriptome analyses revealed that 22 genes were consistently upregulated in H37Rv compared to H37Ra, with significant enrichment in lipid metabolism and cell membrane function pathways (8). Proteomic studies have identified differential protein abundance between H37Rv and H37Ra, particularly in fatty acid biosynthesis and bacterial secretion systems (9, 10). Hiwa Mamatlen *et al.* have further characterized a set of 19 membrane and lipoproteins with higher abundance in H37Rv and 10 proteins with higher abundance in H37Ra (11). In addition, Jhingan *et al.* provided a broader comparative analysis across multiple strains, identifying proteins associated with virulence and drug resistance (12). Despite these findings, the precise mechanisms driving these differences remain unclear.

Small RNAs (sRNAs), non-coding RNA ranging from 50 to 500 nucleotides, have emerged as pivotal regulators in bacterial pathogenicity. These molecules modulate mRNA translation and stability by base-pairing with target mRNAs, enabling bacteria to adapt to stress and host environments (13, 14). Advances in RNA sequencing have identified numerous sRNAs in *M. tuberculosis* (15, 16, 17, 18), many of which display distinct expression patterns under stress conditions, such as exposure to patient serum or plasma, macrophage infection, oxidative stress, pH fluctuations, hypoxia, iron limitation, and starvation. These findings suggest that sRNAs are implicated in processes like latency, growth, infection, and regulatory adjustments (19, 20, 21, 22, 23, 24, 25), and they may play pivotal roles in regulating critical biological processes in *M. tuberculosis*. For instance, *Mcr7* modulates the twin-arginine translocation (Tat) protein secretion system by targeting *tatC* mRNA (26), while *MrsI* (*ncRv11846*) influences iron homeostasis by interacting *bfrA* (27). Additionally, 47 genes have been delineated as potential direct targets of the 6C sRNA, with 15 of these validated using *in vivo* translational *lacZ* fusion systems (28). Despite these advances, the differential expression of sRNAs and their regulatory networks in the virulent H37Rv and attenuated H37Ra strains remains unexplored.

In this study, we integrate label-free quantitative proteomics, parallel reaction monitoring (PRM), and sRNA-seq analyses to unravel the molecular differences between H37Rv and H37Ra. A novel sRNA, *ASpks2*, was identified as a key regulator of the *polyketide synthase 2* (*pks2*) pathway, a critical determinant of lipid metabolism and virulence. By establishing the regulatory relationship between *ASpks2* and *pks2*, this research provides new insights into the molecular mechanisms driving *M. tuberculosis* survival in macrophages and offers potential avenues for therapeutic intervention.

## Results

### Genotypic and phenotypic characterization of *M. tuberculosis* H37Rv and H37Ra

**Figure 1:**
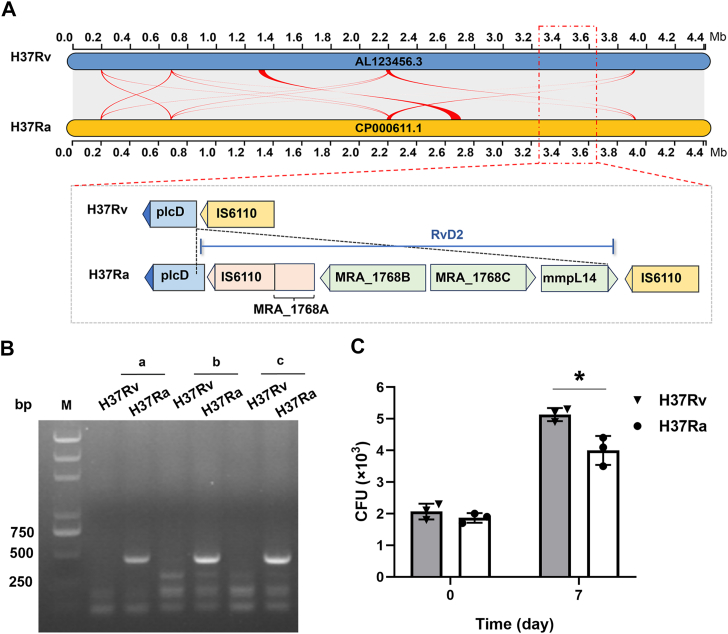
**Phenotypic identification of *M. tuberculosis* H37Rv and H37Ra.***A*, comparison of Progressive Mauve analysis between H37Rv and H37Ra genomes. *B*, PCR analysis RvD2 region in H37Rv and H37Ra. Three fragments about 400 bp in RvD2 (MRA_1768B, MRA_1768C and mmpL14) were selected, and primers were designed. *C*, bacterial counts from H37Rv- and H37Ra-infected THP-1 macrophages at 0 and 7 days post-infection. Results represent the mean ± SD from three independent biological replicates. The individual data points displayed represent the mean of technical replicates from each experiment. *p* values were determined using an unpaired two-tailed Student's *t* test (ns: no significance, ∗*p*< 0.05).

*M. tuberculosis* H37Rv, the virulent reference strain, and H37Ra, its attenuated counterpart, serves as pivotal models for dissecting *M. tuberculosis* virulence mechanisms. The attenuation of H37Ra results from specific genomic mutations accumulated during *in vitro* passage, providing a unique opportunity to study the molecular basis of pathogenicity. Comparative genomic analysis revealed a high degree of similarity between H37Rv and H37Ra genomes, with key distinctions, including 53 insertions, 21 deletions, and 76 strain-specific single nucleotide variants (SNVs) in H37Ra (Fig. 1*A*) (5). It was reported that the H37Ra genome has an insertion of approximately 8000 base pairs between the 5′ end of the *plcD* gene and IS6110 (RvD2 region), which is strong evidence for distinguishing between H37Rv and H37Ra strains. PCR amplification using primers designed for RvD2 confirmed its presence in H37Ra but not in H37Rv (Fig. 1*B*). Under alkaline conditions, H37Rv is capable of staining neutral red dye red, unlike H37Ra (Fig. S1*A*). We conducted a comparative analysis of their characteristics *in vitro* culture and discovered that H37Rv exhibited a faster replication rate in 7H9 liquid culture than H37Ra (Fig. S1*B*). Additionally, we monitored their proliferation within macrophage THP-1 cells and found that the intracellular bacillary load was notably higher in H37Rv-infected cells (mean CFU = 5.1 × 10^3^) than in H37Ra-infected cells (mean CFU = 4.0 × 10^3^) at 7 days post-infection (*p*= 0.02) (Fig. 1*C*).

### Quantitative proteomic characterization of H37Rv and H37Ra

**Figure 2:**
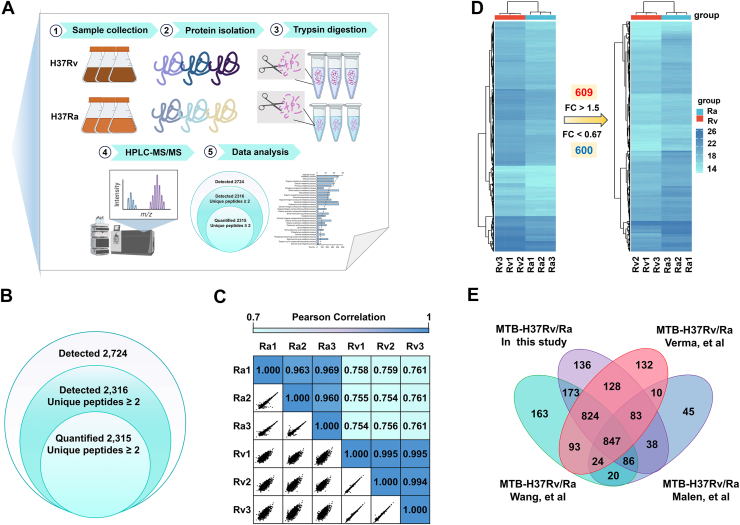
**Quantitative proteomics analysis of *M. tuberculosis* H37Rv and H37Ra.***A*, overall workflow of LFQ proteomic strategies. *B*, Venn diagram of quantified proteins and differentially expressed proteins. *C*, comparison of LFQ-based quantification intensities within three replicates. *D*, heatmaps of quantified intensity of differentially expressed proteins between H37Rv and H37Ra strains (*p*< 0.05). *E*, comparison of the number of quantified proteins in this study with previous literature.

To explore the molecular differences between H37Rv and H37Ra, we compared the proteomic profiles of H37Rv and H37Ra using a label-free quantitative proteomics strategy (Fig. 2*A*). In our assay, a total of 2724 proteins were identified, covering 67% of the annotated H37Rv proteome (4032 proteins, Mycobrowser database) (Fig. 2*B* and Table S2). Of these, 2316 proteins were identified based on the presence of at least two unique peptides, and 2315 proteins were quantifiable. Based on the log_2_-transformed LFQ intensities, we observed very strong correlations among the quantified proteins across replicate samples (R: 0.960–0.995) (Fig. 2*C*), suggesting excellent reproducibility of our MS data. Using a stringent cutoff of fold change > 1.5 and *p*< 0.05, we identified 1209 differentially expressed proteins (DEPs), of which 609 were upregulated and 600 were downregulated. A heatmap visualization further highlighted distinct expression patterns between the two strains, reflecting differences in their relative levels of proteins (Fig. 2*D* and Table S3).

Using the quantitative protein screen of this study as a criterion, our data were systematically compared and analyzed with the previously published proteomics research results of *M. tuberculosis* (9, 10, 11). We found that 136 unique proteins in *M. tuberculosis* were quantified in our experiments, and more than 94.1% of the detected proteins were also included in other H37Rv/H37Ra proteomics datasets. Specifically, there were 847 proteins quantified simultaneously in the four datasets, which are likely to be abundant in the strains and play important functional roles (Fig. 2*E* and Table S4).

### Functional enrichment analysis of DEPs

**Figure 3:**
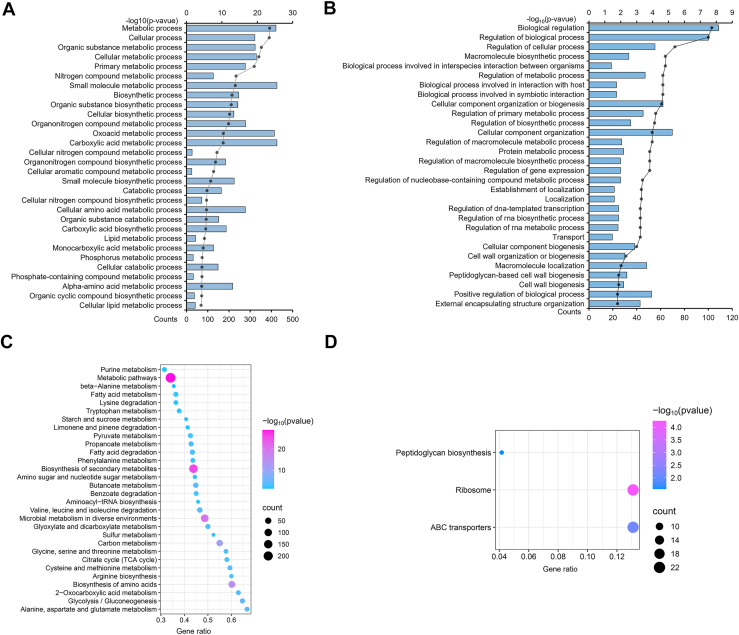
**Functional enrichment analysis of DEPs.***A*, GO enrichment analysis of upregulated DEPs according to biological processes. *B*, GO enrichment analysis of down-regulated DEPs according to biological processes. *C *and* D*, up- and downregulated proteins of KEGG enrichment.

To investigate the biological roles of DEPs, we conducted Gene Ontology (GO) enrichment analysis, classifying proteins based on biological processes, molecular functions, and cellular components. In biological process enrichment analysis, upregulated proteins were enriched in metabolic, cellular, and biosynthesis processes, while downregulated proteins showed enrichment in bioregulation and macromolecular biosynthesis (Fig. 3, *A* and *B*). For molecular functions, upregulated proteins focused on catalytic activity, binding (iron/organic heterocycles), and oxidoreductase activity, whereas downregulated proteins were mainly ribosomal structural components. In cellular localization, upregulated proteins were in cytoplasm, cell wall, and membrane, while downregulated proteins localized to membraneless organelle (Fig. S2 and Table S5). KEGG analysis showed upregulated proteins were involved in purine, β-alanine, and fatty acid metabolism, while downregulated proteins were enriched in ribosome (Fig. 3, *C* and *D*). These suggested H37Rv prioritized energy metabolism for growth, whereas H37Ra focused on regulatory adaptation and protein synthesis. Notably, catalytic/redox activities are key in lipid metabolism. Lipid enzymes (*e.g.*, fatty acid synthases) localize to cytoplasm, while membrane proteins may mediate phospholipid metabolism/transport. *β*-alanine is a precursor of pantothenic acid (CoA component), essential for fatty acid metabolism. Thus, H37Rv likely enhances fatty acid metabolism to acquire energy, stabilize biofilms, or evade immunity.

### Validation of proteomic data by Western blotting and PRM analysis

**Figure 4:**
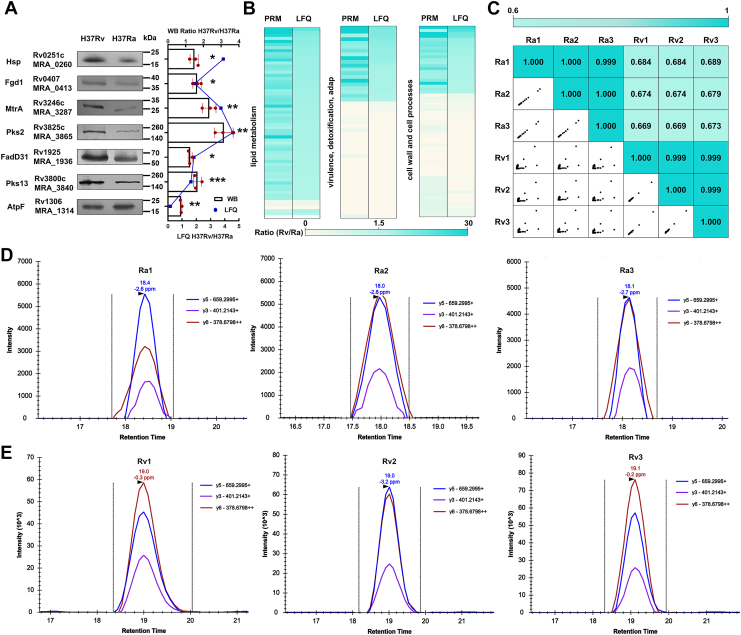
**Verification of the proteomic data from H37Rv and H37Ra.***A*, Western blotting analysis of protein expression levels for seven representative DEPs. Total proteins were extracted and separated by SDS-PAGE. To ensure equal loading, a duplicate gel was run under identical conditions and stained with Coomassie *Blue*. The intensity of total protein per lane was quantified by densitometric scanning, and this was used to normalize the loading volume for Western blot analysis. The proteins were resuspended in 1.5 × loading buffer and subjected to Western blotting using the indicated antibodies. Molecular weight markers (kilodaltons) are indicated. The *left panel* shows a representative blot from three independent experiments. The *right panel* presents the quantification of target protein levels in H37Rv relative to H37Ra. Pixel densities were quantified using ImageJ, and data from independent replicates are presented as mean ± SD (n = 3). The *p* values were determined by unpaired two-tailed Student's *t* test. (∗*p*< 0.05, ∗∗*p*< 0.01, ∗∗∗*p*< 0.001) *B*, heatmaps showing the expression levels of the DEPs selected for validation by PRM. *C*, correlation analysis of converted peak areas for selected DEPs in three groups of biologically replicated samples. *D *and* E*, chromatograms of the extracted ion chromatograms (XICs) of representative peptides of the Pks2 protein from strains H37Rv and H37Ra.

To validate our proteomic results, we performed Western blotting on seven selected DEPs. The results confirmed the upregulation of known virulence factors, including Rv0251c (Hsp) and Rv3246c (MtrA) in H37Rv. Additionally, proteins involved in fatty acid synthesis, such as Rv3825c (Pks2), Rv1925 (FadD31), Rv3800c (Pks13), and Rv0407 (Fgd1) were also upregulated, while Rv1306 (AtpF) was downregulated. These results were consistent with quantitative proteomics results, confirming the reliability of the proteomics data (Fig. 4*A*). We further validated 177 DEPs through PRM analysis, confirming expression changes for 160 proteins with a fold change > 1.5 and *p*< 0.05 (Table S6). As shown in Figure 4*B*, these DEPs were categorized into three functional groups: lipid metabolism, virulence, detoxification, adaptation, and cell wall and cell processes, based on the functional classification of the Mycobrowser database. Heatmaps demonstrated consistent expression patterns between PRM and quantitative proteomics data. Furthermore, to assess the correlation between PRM-based protein expression and quantitative proteomics data, we performed a Pearson correlation analysis based on the peak area of each peptide in its biological replicates. Based on the scatter plots, the strong linear correlation observed indicated high reproducibility between replicates (Fig. 4*C*). Observe that the expression levels of lipid metabolism-related proteins were significantly elevated in the H37Rv strain, such as KasA, FadD32, and Pks13, which are involved in mycolic acid synthesis (29), as well as key proteins in the sulfolipid synthesis pathway, including Pks2 and FadD23 (30). Figure 4*D* shows the extracted ion chromatograms of representative peptides of the Pks2 protein in the H37Rv and H37Ra strains. These findings reveal the important role played by lipid metabolism pathways in the regulation of bacterial virulence.

### Protein–protein interaction network (PPI)

**Figure 5:**
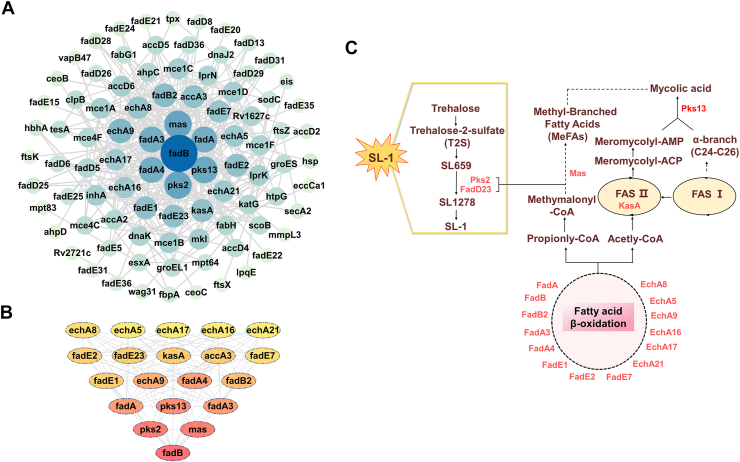
**Network analysis of validated DEPs.***A*, visualized PPI network of validated DEPs. *B*, *top*-ranked network cluster in the PPI network based on Cytohubba plugin (*Top* 20 DEPs). *C*, illustrations of the 20 differentially expressed proteins involved in lipid and fatty acid metabolism pathways in *M. tuberculosis*. *Red* shows up-regulated proteins.

To further obtain the potential biological roles of the DEPs, a total of 160 PRM-validated DEPs were integrated into the STRING database. We successfully constructed a protein–protein interaction (PPI) network containing 91 proteins with high confidence scores (>0.7) (Fig. 5*A*). Using the CytoHubba plugin, we identified the most important protein core modules in the network, comprising 20 proteins, namely, Rv0860 (FadB), Rv3825c (Pks2), Rv3800c (Pks13), Rv2940c (Mas), Rv1074c (FadA3), Rv0859 (FadA), Rv0131c (FadE1), Rv0154c (FadE2), Rv3140 (FadE23), Rv0400c (FadE7), Rv1070c (EchA8), Rv0675 (EchA5), Rv3039c (EchA17), Rv2245 (KasA), Rv1071c (EchA9), Rv2831 (EchA16), Rv3774 (EchA21), Rv0468 (FadB2), Rv3285 (AccA3), and Rv1323 (FadA4) (Fig. 5*B*). Most of these core proteins are acyl-CoA-related enzymes and polyketide synthases, which are involved in various metabolic pathways, such as fatty acid metabolism and lipid synthesis pathways (Fig. 5*C*). SL-1, an abundant sulfate ester specifically expressed in pathogenic mycobacteria, mainly exists on the outer membrane of *M. tuberculosis*, and its levels correlate with the virulence of the strain (31). Interestingly, genes involved in the SL-1 biosynthesis pathway *pks2* and *fadD23* showed significant upregulation in H37Rv, further highlighting their potential role in *M. tuberculosis* virulence.

### Prediction of virulence-associated sRNAs

**Figure 6:**
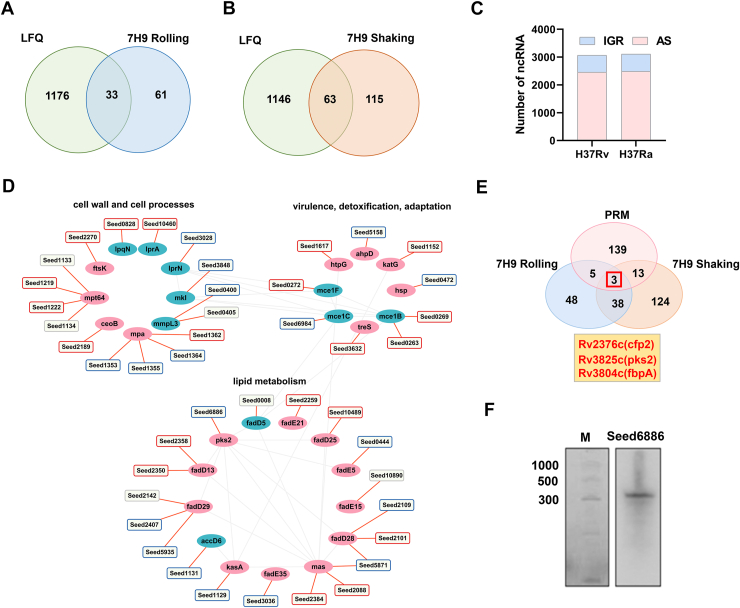
**Analysis of sRNAs network.***A and B*, overlap of DEGs and DEPs under shaking and rolling conditions. *C*, distribution of sRNAs in *M. tuberculosis* H37Rv and H37Ra. *D*, the network map of sRNAs involved in the regulation of virulence. The squares represent sRNAs and ellipses show proteins, *red* means “Up”, *blue* means “Down”, *grey* means no significance. *E*, overlapping of PRM-validated proteins with differential expressed genes in the GSE3999 dataset. *F*, identification of sRNA presence by Northern blotting.

To investigate whether the extensive proteomic remodeling observed between H37Rv and H37Ra under our static culture conditions could be traced to transcriptional regulation, we turned to publicly available RNA-seq datasets. We reanalyzed data from a previous study by Gao *et al.* (8). that compared H37Rv and H37Ra transcriptomes under two different growth conditions (shaking and rolling) in 7H9 medium. While the original study reported differentially expressed genes (DEGs), the gene lists were not provided. Our reanalysis identified 94 and 178 DEGs under rolling and shaking conditions, respectively (Table S7). We reasoned that genes showing consistent transcriptional differences between strains across diverse growth environments might represent core, condition-independent regulatory signatures. Therefore, we compared these transcriptomic datasets, derived from shaking and rolling cultures, with our list of 1209 differentially abundant proteins (DEPs) obtained from static cultures. This comparison revealed that 33 of the shaking-responsive genes and 63 of the rolling-responsive genes also showed significant differential expression at the protein level in our system (Fig. 6, *A* and *B*).

Interestingly, for a subset of genes, the direction of change at the transcript and protein levels was not positively correlated, with 18 genes showing a clear negative correlation. This observed discordance between the transcriptomic and proteomic datasets suggests that, while some transcriptional programs are robust to changes in culture conditions, post-transcriptional regulatory mechanisms likely play a substantial role in shaping the final proteomic landscape in mycobacteria.

sRNAs have been found to play key roles in post-transcriptional regulation in a wide range of bacteria, influencing critical cellular processes and helping bacteria in adapting to environmental changes (32, 33, 34). We therefore hypothesized that strain-specific differences in sRNA expression could underlie, at least in part, the divergence in protein expression between H37Rv and H37Ra. Most sRNAs exert their regulatory effects by base-pairing with target mRNAs, thereby influencing transcription efficiency, mRNA stability, or translation. To systematically identify non-coding RNAs in the two strains, we performed sRNA-seq analysis on H37Ra and integrated these data with previously published H37Rv sRNA datasets from Wang *et al.* This analysis identified a total of 3066 non-coding RNAs in H37Rv, including 2454 antisense (AS) and 612 intergenic RNAs (IGR) (Fig. 6*C*). For H37Ra, we identified 3108 non-coding RNAs, comprising 2487 AS RNAs and 621 IGR RNAs (Fig. 6*C* and Table S8). To explore potential regulatory relationships between sRNAs and strain-differential proteins, we integrated these predicted sRNAs from H37Rv and H37Ra strains with the PRM validated interaction network of DEPs, suggesting that these proteins could potentially serve as targets for these identified non-coding RNAs, as shown in Figure 6*D*. These proteins were categorized into three main functional groups based on their GO functions listed in the Mycobrowser database. Notably, sRNAs appear to be involved in the regulation of various DEPs, with a focus on lipid metabolism, cell wall and cell processes, virulence, adaptation, and detoxification. Furthermore, one gene may be targeted by one or more sRNAs, which exhibit differential expression between H37Rv and H37Ra, highlighting the complexity of the non-coding RNA regulatory landscape in *M. tuberculosis*. For example, the Rv2115c (*mpa*) was targeted by four different sRNAs.

To prioritize candidates for experimental validation, we further compared PRM-validated DEPs with genes that were differentially expressed at the transcript level under both shaking and rolling growth conditions (Table S7). This integrative analysis identified three genes, including Rv2376c (*cfp2*), Rv3825c (*pks2*), and Rv3804c (*fbpA*), which consistently showed significant differences at both the protein and the transcript levels (Fig. 6*E*). Among these candidates, *pks2* was selected as a representative gene for further investigation, owing to its established role in lipid metabolism and virulence-associated processes in *M. tuberculosis*. Our sRNA-seq analysis revealed a candidate antisense RNA (*Seesd6886*) located antisense to the *pks2* gene in *M. tuberculosis* (Fig. 6*D*); its expression showed a negative correlation with that of *pks2*. We subsequently validated the existence of this ncRNA by Northern blot analysis and successfully verified the presence of this ncRNA in the H37Rv strain (Fig. 6*F*). To define its transcript boundaries, 3' RACE analysis was performed by the ligation of an adapter to the 3' hydroxyl group of H37Rv total RNAs, which defined the 3’ end of this ncRNA and supported its antisense genomic localization relative to *pks2* (Fig. S3). Given its proximity to *pks2* in the genome, we designated this ncRNA *ASpks2*, the AS RNA of *pks2*.

Collectively, this integrative analysis positioned *ASpks2* as a functionally representative case within the broader sRNA regulatory network distinguishing H37Rv and H37Ra. We selected it for mechanistic validation, not as a singular determinant of strain divergence, but as a tractable model that directly links system-level omics disparities in lipid metabolism and virulence to a concrete post-transcriptional regulatory mechanism.

### Pks2 is the major target of *ASpks2*

**Figure 7:**
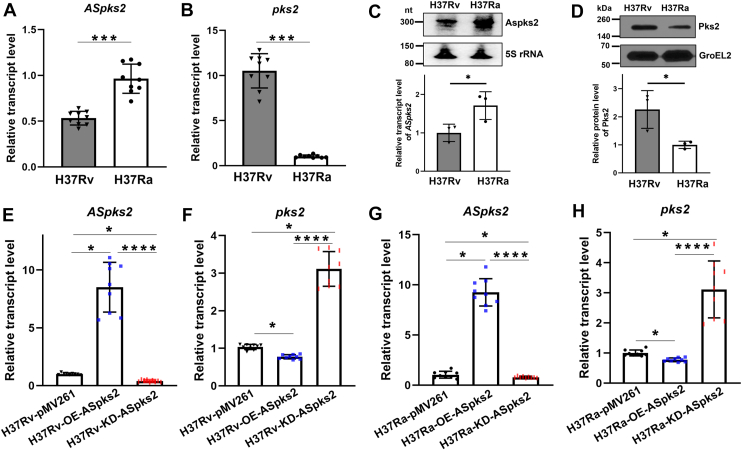
**Characterization of *pks2* as the major *ASpks2* target.***A and B*, qRT-PCR analysis of *ASpks2* or *pks2* from H37Rv and H37Ra. *C*, northern blot analysis of the *ASpks2* transcript in H37Rv and H37Ra. 5S rRNA was used as a loading control. Molecular weight markers (nucleotide) are indicated. A representative blot from three independent experiments is shown. The graph below shows quantification of *ASpks2* RNA levels in H37Rv relative to H37Ra. Statistical significance was determined using a two-tailed unpaired Student's *t* test. *D*, western blotting of Pks2 and GroEL2 in the two strains. GroEL2 was used as control for the bacterial integrity of each sample. Molecular weight markers (kilodaltons) are indicated. The graph below shows quantification of Pks2 protein levels in H37Rv relative to H37Ra. *E *and* F*, qRT-PCR analysis of *ASpks2* and *pks2* in the H37Rv-pMV261, H37Rv-OE-*ASpks2* and H37Rv-KD-*ASpks2* strains. *G *and* H*, qRT-PCR analysis of the *ASpks2* and *pks2* in the H37Ra-pMV261, H37Ra-OE-*ASpks2* and H37Ra-KD-*ASpks2* strains. One-way analysis of variance (ANOVA) was performed, followed by Tukey's test for pairwise comparisons. Data are presented as mean ± SD from at least three biological replicates. (∗*p*< 0.05, ∗∗∗*p*< 0.001, ∗∗∗∗*p*< 0.0001).

Notably, qPCR and Northern blot analyses showed that *ASpks2* transcription was lower in H37Rv compared to H37Ra (Fig. 7, *A* and *C*), whereas qPCR and Western blot analyses of *pks2* mRNA and protein levels were higher in H37Rv (Fig. 7, *B* and *D*). These observations led us to hypothesize that *ASpks2* negatively regulates *pks2* expression.

To further determine whether *ASpks2* specifically targets *pks2*, we constructed the overexpression and knockdown strains of *ASpks2* by cloning the *ASpks2* fragment and its complementary paired fragment into the pMV261 vector. Subsequently, the expression levels of *ASpks2* were verified by qPCR and Northern blotting (Figs. 7*E* and S4*B*). In line with our hypothesis, both the mRNA and protein levels of Pks2 were significantly reduced in the H37Rv-OE-*ASpks2* strain, while showing significant upregulation in the H37Rv-KD-*ASpks2* strain (Figs. 7*F* and S4*C*). Parallel experiments conducted in the avirulent H37Ra strain, involving the overexpression and knockdown of *ASpks2*, yielded a similar inverse correlation between *ASpks2* and *pks2* expression (Fig. 7, *G* and *H*). Importantly, the protein levels of PapA1, which is downstream of Pks2, remained unchanged in the H37Rv-OE-*ASpks2* strain or the KD-*ASpks2* strain, confirming the specificity of *ASpks2* in targeting *pks2* (Fig. S4, *A* and *C*). Therefore, we concluded that *ASpks2* functions as a negative regulator of *pks2*, suppressing its transcription and reducing its protein abundance.

### *ASpks2* promotes H37Rv growth in human macrophages

**Figure 8:**
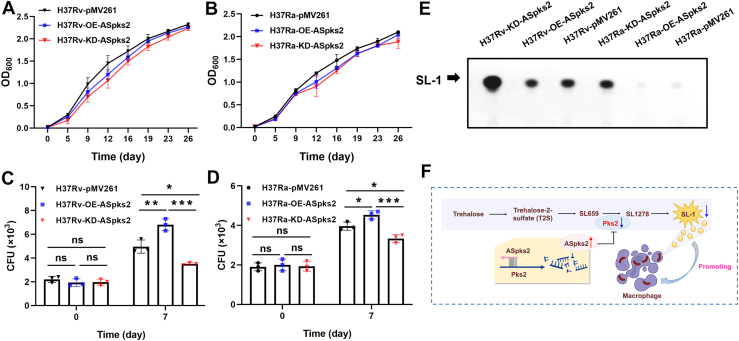
***ASpks2* promotes the growth of H37Rv in human viable THP-1 macrophages.***A*, growth curves of the H37Rv-pMV261, H37Rv-OE-*ASpks2* and H37Rv-KD-*ASpks2* strains in 7H9 medium. Bacterial growth was monitored by measuring OD_600_ at the indicated time points. Under static culture conditions, all cultures were inoculated with an initial OD_600_ of 0.1. *B*, growth curves of the H37Ra-pMV261, H37Ra -OE-*ASpks2* and H37Ra -KD-*ASpks2* strains in 7H9 medium. *C *and* D*, Intracellular loads of H37Rv (pMV261, OE-*ASpks2*, and KD-*ASpks2*) or H37Ra (pMV261, OE-*ASpks2*, and KD-*ASpks2*) were assessed at 0 h and 7 days post-infection. Data are presented as mean ± SD from three independent experiments. Statistical significance was determined by One-way analysis of variance (ANOVA) followed by Tukey's test (ns: no significance, ∗*p*< 0.05, ∗∗*p*< 0.01, ∗∗∗*p*< 0.001). *E*, TLC analysis of crude extracts from ^14^C-propionic acid-labeled cultures *M. tuberculosis* H37Rv (pMV261, OE-*ASpks2*, and KD-*ASpks2*) or H37Ra (pMV261, OE-*ASpks2*, and KD-*ASpks2*). *Blackarrowhead*, position of SL-1. *F*, potential regulatory role of sRNA *ASpks2* in *M. tuberculosis*.

To explore the biological function of *ASpks2* in *M. tuberculosis* H37Rv, we investigated the phenotypic differences among the control H37Rv-pMV261, H37Rv-OE-*ASpks2*, and H37Rv-KD-*ASpks2* strains. Under static condition, growth curves showed no significant differences among the strains (Fig. 8*A*). Notably, the H37Rv-OE-*ASpks2* strain showed visible sedimentation at the bottom of the flask, whereas the control and H37Rv-KD-*ASpks2* strains remained more uniformly dispersed in 7H9 liquid medium (Fig. S5). These observations indicated that the sedimentation phenotype reflects altered physical aggregation properties rather than growth defects. We next investigated the potential role of *ASpks2* in virulence by performing *ex vivo* infections. Human macrophage THP-1 cell lines were infected with negative control, H37Rv-OE-*ASpks2* strain, or H37Rv-KD-*ASpks2* strain, and intracellular growth was assessed. On day 7 post-infection, CFU counts of bacteria within adherent viable cells revealed a significant increase in colony numbers for the H37Rv-OE-*ASpks2* strain, whereas the H37Rv-KD-*ASpks2* strain showed a marked reduction (Fig. 8*C*). We also evaluated the impact of *ASpks2* in the H37Ra background and found that its overexpression or knockdown similarly increased or decreased bacterial survival, respectively (Fig. 8, *B* and *D*). Furthermore, we assessed the levels of SL-1 and found that its abundance was significantly diminished in the H37Rv-OE-*ASpks2* strain but markedly increased in the H37Rv-KD-*ASpks2* strain. A similar pattern of SL-1 expression was also observed in the corresponding H37Ra engineered strains (Fig. 8*E*).

Our findings support a model where *ASpks2* mitigates host cell damage by downregulating *pks2* expression. This protective effect may stem from the inhibition of SL-1 synthesis, leading to alterations in the *M. tuberculosis* cell wall composition and contributing to a more stable host-pathogen relationship (Fig. 8*F*). This mechanism highlights the critical role of *ASpks2* in modulating bacterial virulence and enhancing intracellular survival.

## Discussion

sRNAs are critical regulators of bacterial pathogenicity, playing essential roles in processes such as host immune evasion, stress adaptation, and metabolic reprogramming. By integrating proteomic and transcriptomic profiles of the virulent H37Rv and attenuated H37Ra strains, this study aimed to decipher the regulatory architecture underlying their distinct pathogenic phenotypes. This systems-level comparison served as the foundation for identifying candidate regulators, among which we functionally characterized the antisense sRNA *ASpks2* as a representative case linking post-transcriptional control to a defined virulence pathway.

Specifically, *ASpks2* was shown to downregulate *pks2* expression. Overexpression of *ASpks2* led to significant reductions in *pks2* expression, while knockdown of *ASpks2* resulted in its upregulation (Fig. 7*F*). In H37Rv, using specific antibodies, we confirmed that *ASpks2* selectively downregulated Pks2 without influencing the protein expression of the downstream gene PapA1. These findings suggest that *ASpks2* specifically targets *pks2*, leading to reduced protein levels. Notably, comparative genomic analysis revealed that the *pks2* gene and its surrounding locus are identical between the virulent H37Rv and attenuated H37Ra strains. This conservation strengthens the conclusion that the differential expression of *pks2* observed between these strains is likely attributable to differential expression or activity of *ASpks2* itself, rather than genetic divergence at the *pks2* locus. The genetic conservation of the *pks2* locus between the two strains enabled us to specifically interrogate the functional consequences of manipulating *ASpks2* levels in H37Ra. We generated H37Ra strains with *ASpks2* overexpression and knockdown, confirming the expected regulatory changes. As shown in Figure 7, *G* and *H*, knockdown of *ASpks2* in the attenuated H37Ra strain led to a corresponding upregulation of *pks2*, while its overexpression suppressed *pks2* levels. These results, together with the differential expression levels of *ASpks2* and *pks2* observed between H37Rv and H37Ra using qPCR, Northern blot, and Western blot, demonstrate that the differential activity of *ASpks2* itself is the primary determinant of the divergent *pks2* expression between H37Rv and H37Ra.

In addition, we found that overexpression of *ASpks2* significantly affected mycobacterial aggregation behavior and enhanced the survival of *M. tuberculosis* H37Rv within human THP-1 macrophages, as evidenced by increased CFU counts. Under static conditions, we observed the visible sedimentation in the H37Rv-OE-*ASpks2* strain (Fig. S5). Previous studies have shown that WhiB3 regulates *pks2* and SL-1 biosynthesis and that disruption of this lipid regulatory pathway can influence bacterial aggregation behavior (35). Given that *ASpks2* negatively modulates *pks2* expression, we reasoned that the altered settling behavior observed here may reflect changes in cell-envelope lipid balance that affect inter-bacterial interactions. The increased sedimentation observed under static conditions is consistent with this conceptual framework. However, we interpret this as a phenotypic correlation rather than direct mechanistic evidence. This observation suggests a potential connection between *ASpks2*-mediated regulation and aggregation-related phenotypes, which warrants further investigation.

The gene *pks2* is involved in the synthesis of SL-1, a lipid that is up-regulated during infection of both human macrophages and in mice (35, 36, 37). SL-1 has been proposed to play multiple roles in host physiology, including modulation of cytokine secretion, interference with phagosome maturation, regulation of membrane dynamics, and alteration of host signaling pathways (38, 39, 40, 41). These findings have traditionally positioned SL-1 as a classical virulence-associated lipid that contributes to immune evasion. However, the observation that *ASpks2* overexpression in H37Rv, presumably reducing SL-1 levels, led to enhanced intracellular survival (Fig. 8, *C* and *D*) suggests that the functional role of SL-1 in macrophage infection may be more complex than previously appreciated. Emerging evidence indicates that SL-1 may also participate in activating lysosome-associated host defense pathways (42). For example, SL-1 has been reported to promote host antimicrobial responses through activation of the mTORC1-TFEB axis, thereby enhancing lysosomal biogenesis and phagosome maturation. Within this conceptual framework, *M. tuberculosis* strains lacking *pks2* exhibit reduced delivery to lysosomes and enhanced intracellular survival. In this context, the increased CFU observed for the H37Rv-OE-*ASpks2* strain may reflect reduced SL-1 levels leading to diminished stimulation of host lysosomal defense pathways, thereby creating a more permissive intracellular environment. Conversely, the KD-*ASpks2* strain, with relatively higher SL-1 levels, may induce stronger lysosome-associated responses and thus show lower intracellular bacterial burden.

Importantly, the attenuated H37Ra strain is defective in SL-1 production due to a mutation in the phoP regulator rather than in *pks2* gene itself, highlighting that multiple regulatory layers contribute to lipid-associated phenotypes (36). In this context, *ASpks2* represents an additional post-transcriptional regulatory element capable of modulating the *pks2*–SL-1 axis. The elevated *ASpks2* level in H37Ra may contribute to reduced Pks2 expression and altered lipid balance, potentially participating in the broader phenotypic differences between H37Ra and H37Rv. While SL-1 is unlikely to be the sole determinant of virulence attenuation, our findings support the notion that *ASpks2*-mediated regulation constitutes one component of the multilayered regulatory network underlying strain-specific pathogenic traits.

Beyond to the role of *ASpks2* in regulating *pks2*, our study underscores the broader impact of sRNAs on *M. tuberculosis* pathogenesis. The integration of proteomic and transcriptomic data revealed a complex network of proteins and pathways involved in the bacterium's adaptation to the host environment. Specifically, we observed that upregulated proteins in H37Rv were enriched in metabolic process, fatty acid metabolism, cell wall and membrane components. These findings align with the notion that *M. tuberculosis* employs a multifaceted approach to adapt to host immune pressures (43, 44), and sRNAs such as *ASpks2* serve as crucial regulators of this adaptive response. By modulating key metabolic and virulence pathways, *ASpks2* enables *M. tuberculosis* to fine-tune its virulence profile and maximize its chances of survival within the host.

Furthermore, this study emphasizes the growing recognition of sRNAs as key players in bacterial adaptation and pathogenesis. Similar regulatory roles for sRNAs have been identified in other pathogens, such as *Pseudomonas aeruginosa*, *Staphylococcus aureus*, and *Listeria*, where sRNAs influence both chronic and acute infection regulation as well as immune evasion (45, 46, 47). The conservation of sRNA-mediated regulatory mechanisms across different pathogens suggests that targeting sRNA networks could offer a promising therapeutic strategy for modulating bacterial virulence. Disrupting sRNA regulation could reduce the pathogen’s ability to adapt to the host and enhance immune responses, providing a potential complement to traditional antibiotics, particularly in the context of drug-resistant strains. By targeting multiple virulence factors, this strategy could make it easier for the host immune system to clear the infection.

It is important to emphasize that *ASpks2* is presented here not as the solitary determinant of the extensive phenotypic gap between H37Rv and H37Ra, but as a mechanistically elucidated paradigm. It exemplifies how antisense RNA-mediated fine-tuning of key metabolic enzymes, such as Pks2 in SL-1 synthesis, can directly contribute to strain-specific virulence traits. Our work thus establishes a framework: initial systems biology comparisons between strains highlight regulatory “hotspots,” which can then be mechanistically dissected through focused studies on individual nodes like *ASpks2*. This approach successfully bridges the gap between large-scale “omics” observations and concrete, testable regulatory mechanisms.

Although this study provides valuable insights into the role of *ASpks2* in *M. tuberculosis* virulence, two key areas require further investigation. First, further studies are needed to explore how *ASpks2* interacts with other sRNAs and regulatory proteins to coordinate the broader regulatory network that governs *M. tuberculosis* virulence. Understanding the full scope of regulatory actions of *ASpks2* will provide a more comprehensive understanding of its role in bacterial pathogenesis. Second, investigating the role of *ASpks2* under stress conditions, such as nutrient limitation, hypoxia, and antibiotic exposure, will shed light on its contribution to *M. tuberculosis* survival in latent and chronic infections.

In conclusion, the integrated transcriptomic and proteomic analysis establishes an sRNA regulatory network in *M. tuberculosis*. We identify a new sRNA *ASpks2*, which targets *pks2* and affects the survival of *M. tuberculosis* in human macrophage cells. This study serves as the first proteome-wide analysis of sRNA regulatory networks and provides new clues for the mechanism involved in the virulence of *M. tuberculosis*.

## Experimental procedures

### Biosafety notes

*M. tuberculosis* H37Rv was handled in a biosafety level 3 (BSL-3) laboratory under stringent biosafety protocols to minimize exposure risks. The attenuated H37Ra strain was handled in a BSL-2 environment (48). World Health Organization (WHO) Biosafety Manual for Tuberculosis Laboratories provides a series of safety measures to ensure that the risk of accidental transmission is minimized when conducting experiments related to *M. tuberculosis* (including H37Rv and H37Ra) (49).

### Bacterial strains and culture conditions

*M. tuberculosis* H37Rv and H37Ra were cultured in Middlebrook 7H9 medium supplemented with 10% (v/v) OADC (oleic acid, albumin, dextrose, and catalase) enrichment, 0.5% glycerol, and 0.05% Tween 80 or on 7H10 plates supplemented with 10% OADC. For plasmid-containing strains, 20 μg/ml kanamycin was added. Cultures were grown at 37 °C under static conditions until the logarithmic phase (OD_600_= 0.7–0.8), at which point cells were harvested for downstream RNA and protein extraction.

### Protein extraction and digestion

Bacterial cells were washed twice with ice-cold PBS buffer, resuspended in ice-cold PBS containing 1 × protease inhibitor mixture (Roche Diagnostics Ltd) and 1 mM PMSF. The mixture was processed in a nucleic acid extractor Fastprep-24 (MP Biomedicals) for 35 s at a speed of 6.5 m/s for five cycles as previously described (50). Cell lysates were centrifuged at 12,000*g* for 15 min at 4 °C to remove cellular debris. Supernatant was then filtered through the 0.2 μm syringe filters (Merck Millipore) twice to sterilize lysate. The protein concentration was determined by BCA assay (Beyotime) following the manufacturer’s instructions.

Proteins from three biological replicates were reduced, alkylated, trypsin digested and desalted as described previously (51). Briefly, 100 ug of each group of samples was reduced with 25 mM dithiothreitol (DTT) for 45 min at 37 °C and alkylated with 50 mM iodoacetamide (IAM) for 10 min in the dark. Then, the samples were digested with trypsin to a protein mass ratio of 1:100 at 37 °C for 24 h, followed by the addition of 0.1% (v/v) trifluoroacetic acid (TFA) to terminate the digestion. The digests were finally desalted using a self-packed C_18_ STAGE column and dried using a vacuum centrifuge.

### LC-MS/MS analysis

After dissolving with 1% formic acid (FA), peptides were separated on an online nanoEASY1200 HPLC system (Thermo Fisher Scientific) coupled with an Orbitrap Q Exactive HF-X mass spectrometer (Thermo Fisher Scientific) on a 75 μm × 15 cm analytical column (Thermo Fisher Scientific). The nanopump provided a flow-rate of 300 nl/min and was operated under gradient elution conditions, using 0.1% FA as buffer A, and 0.1% FA in 90% ACN as buffer B. Gradient elution was performed according the following scheme: 1 min of gradient from 4 to 8% buffer B, 34 min of gradient from 8 to 13% buffer B, 26 min of gradient from 13 to 18% buffer B, 25 min of gradient from 18 to 25% buffer B, 19 min of gradient from 25 to 35% buffer B, 10 min of gradient from 35 to 40% buffer B, 1 min of gradient from 40 to 100% buffer B, and a final 9 min of 100% buffer B to wash the column. Subsequently, the eluted peptides were ionized and detected using the Orbitrap Q Exactive HF-X mass spectrometer (Thermo Fisher Scientific). MS data were collected in data-independent acquisition (DIA) mode using 100 variable windows covering a mass range of 350 to 1200 at a resolution of 60,000. The normalized AGC target was set to 3E6 and the maximum injection time was 50 ms. Following every survey scan, the m/z range of 400 to 1000 was acquired at 30,000 resolution with 6 m/z of isolation window for DIAscan. Precursor ions were selected for fragmentation by high-energy collision dissociation with a normalized collision energy of 30%. The normalized AGC target was set to 2E5 and the maximum injection time was set to auto.

### Database search and protein quantification

LC-MS raw files were processed using DIA-NN software (version 1.8.1) (52) using a library free workflow against the H37Rv (GCA_000195955.2) and H37Ra (GCA_000016145.1) databases concatenated with common contaminants. “FASTA digest for library free search/library generation” and “Deep learning spectra, RTs and IMs prediction” options were used for precursor ion generation. Two missed cleavages were allowed for trypsin. Carbamidomethylation (Cys) was set as a fixed modification, whereas dynamic modifications were set as oxidation (Met) and acetylation (Protein N-terminal). Maximum number of variable modifications was set to 1. Peptide length was set to 7 to 30 amino acids, and the precursor charge range was restricted to 1 to 4. The FDR at the precursor, peptide and protein levels was set to 1%. Other parameters were kept at their default values in DIA-NN software. Quantification was performed by LFQ algorithm. LFQ intensities were extracted from the DIA-NN report file “report.pg_matrix.tsv”. Data analysis and statistical evaluation were done with Microsoft Excel. Only proteins identified by at least two unique peptides were reported. LFQ intensities had to be detected in two biological replicates in one of the groups were included in the relative quantitative analysis. Missing values in the third biological replicate were estimated using the default parameters for normal distribution in Perseus (53). The number of unique and razor peptides for protein quantification was determined using the default quantification strategy of the DIA-NN software. The total LFQ intensities of all identified proteins were normalized and the average LFQ intensity for each biological sample group was derived by calculating the arithmetic mean. Statistical assessments were performed using a two-sample Student's *t* test, and differentially expressed proteins (DEPs) were defined as fold change (FC) > 1.5 or < 0.67 with *p*-value < 0.05.

### DIA and parallel reaction monitoring (PRM) experiments

For DIA-based experiments, the digested samples were analyzed on an Ultimate 3000 HPLC system (Dionex) coupled with an Orbitrap Exploris 240 mass spectrometer (Thermo Fisher Scientific) on a 75 μm × 15 cm analytical column (Thermo Fisher Scientific). Peptides were eluted using a 135 min gradient of buffer B as follows: 0 to 5 min, 2% solvent B at a flow rate of 400 nl/min; five to 5.5 min, 2% solvent B at a flow rate of 300 nl/min; 5.5 to 11 min, 2 to 3% solvent B at a flow rate of 300 nl/min; 11 to 40 min, 3 to 7.5% solvent B at a flow rate of 300 nl/min; 40 to 102 min, 7.5 to 18% solvent B at a flow rate of 300 nl/min; 102 to 117 min, 18 to 25% solvent B at a flow rate of 300 nl/min; 117 to 122 min, 25 to 33% solvent B at a flow rate of 300 nl/min; 122 to 127 min, 33 to 55% solvent B at a flow rate of 300 nl/min; 127 to 128 min, 55 to 99% solvent B at a flow rate of 400 nl/min; 128 to 135 min, 99% solvent B at a flow rate of 400 nl/min. The eluted peptides were ionized and detected using the Orbitrap Exploris 240 mass spectrometer (Thermo Fisher Scientific). MS data were collected in data-independent acquisition (DIA) mode using 100 variable windows covering a mass range of 350 to 1200 at a resolution of 120,000. The normalized AGC target was set to 300% and the maximum injection time was 50 ms. Following every survey scan, the m/z range of 400 to 1000 was acquired at 30,000 resolution with 6 m/z of isolation window for DIAscan. Precursor ions were selected for fragmentation by high-energy collision dissociation with a normalized collision energy of 30%. The normalized AGC target was set to 200% and the maximum injection time was set to 100 ms. All raw files were processed using DIA-NN software (version 1.8.1) with the same parameters as described above (“Database search and protein quantification”). The peptide identification results were imported into Skyline software (54) as a spectral library. Based on the spectral library results, the transition lists of the target tryptic peptides were generated and used for PRM analysis.

For PRM experiments, the digested samples were analyzed on an Ultimate 3000 HPLC system (Dionex) coupled with an Orbitrap Exploris 240 mass spectrometer (Thermo Fisher Scientific) on a 75 μm × 15 cm analytical column (Thermo Fisher Scientific). MS data collection was performed in PRM acquisition mode with the same gradient elution conditions and MS parameters as described in the DIA-based experiments. All PRM data were processed using Skyline software as previously described (55). Briefly, to avoid false peptide identification, three transitions per peptide were at least recommended to ensure enough transitions and to maintain the selectivity for reliable quantification. Peaks were manually checked for correct integration, and the area under the curve (AUC) of targeted peptide was obtained from the summation of the AUC for each transition peak. The abundance of each peptide was normalized based on the average abundance of each protein. For protein quantification, the average of the target peptide abundances was used to calculate the fold change between samples.

## Bioinformatics analysis

Gene Ontology (GO) and Kyoto Encyclopedia of Genes and Genomes (KEGG) pathway enrichment analyses were performed using STRING and DAVID databases (56, 57, 58). The GO terms and KEGG pathways were considered significantly enriched when they met a *p*-value < 0.05. Using the Mycobrowser database for functional category (59). Genome analysis was performed using TBtools software (60). The heatmaps were generated using R software (4.2.1) and the ComplexHeatmap package (2.13.1). Protein-protein interaction (PPI) network analysis was performed using the STRING database. The network was visualized by Cytoscape software (v3.9.1) and further analyzed for densely connected regions using the cytoHubba plugin in Cytoscape (61). Correlation values were calculated using the Pearson correlation coefficient test.

### RNA extraction and sRNA-seq

The small RNA (sRNA) sequencing data for the H37Rv strain were retrieved from previous study (16). For H37Ra, we collected the bacterial cells that grown in the same cultivation condition with H37Rv strain. The cells were lysed using a bead mill, and the resulting supernatant was subjected to centrifugation. Further purification involved two chloroform extractions, isopropanol precipitation, and washing with 80% ethanol to ensure high RNA purity. The extracted RNA was treated with 10 U DNase I (Fermentas) at 37 °C for 30 min to prevent genomic DNA contamination, followed by additional purification steps. A total of 10 μg of RNA was further processed to deplete rRNA using the MICROBExpress Kit (Ambion) according to the manufacturer’s instructions. The RNA was fractionated *via* urea polyacrylamide gel electrophoresis into three size classes: Fraction 1 (18–40 nt), Fraction 2 (40–80 nt), and Fraction 3 (80–140 nt). The remaining sample, consisting of rRNA-depleted RNA, was fragmented to ∼140 nt using divalent cations at high temperatures. Then, strand-specific cDNA libraries were prepared following the Illumina TruSeq protocol, as previously described (62). For fractions one and two samples, cDNA constructs were prepared by reverse transcription followed by low-cycle PCR amplification. PCR products were collected by gel purification and sequenced on the Illumina Genome Analyzer II (Illumina) platform. For fractions three and four samples, RNA libraries were constructed using the TruSeq mRNA kit (Illumina) according to the manufacturer’s instructions and sequencing was performed on an Illumina Genome Analyzer II (Illumina) platform.

Raw reads were processed as described by Wang *et al.* (16). Briefly, low-quality reads and adapter sequences were removed using Trimmomatic tools (version 0.32) (63) with the following criteria: 1) Removal of adapter and primer-matched sequences; 2) Trimming leading and trailing bases with a Phred33 score <3; 3) Applying a sliding window of four bases, cutting reads when the average base quality dropped below 15; 4) Discarding reads shorter than 16 bases after trimming. The clean reads were then aligned to the *M. tuberculosis* H37Rv reference genome (NC_000962.3) or H37Ra reference genome (NC_009525.1) using Bowtie2 (64) with default parameters. The strand-specific read coverage at each genomic base were generated using SAMtools (65) and BEDTools (66). Data were visualized in the Integrated Genome Browser (IGB) (67) to assess sequence coverage at each genomic position. In addition, BLAST was used to compare sequences and match sRNAs between H37Rv and H37Ra strains (68).

### Production of polyclonal antibodies

Anti-Hsp (Heat shock protein Hsp), Fgd1 (F420-dependent glucose-6-phosphate dehydrogenase Fgd1), MtrA (Two component sensory transduction transcriptional regulatory protein MtrA), Pks2 (Polyketide synthase Pks2), FadD31 (Probable acyl-CoA ligase FadD31), Pks13 (Polyketide synthase Pks13), and AtpF (Probable ATP synthase B chain AtpF) polyclonal antibodies were produced and purified *via* affinity chromatography by ABclonal (Wuhan, China). Briefly, the full-length or C-terminal region cDNA of H or AtpF was amplified, PCR products were cloned into the pGEX-4T-1 expression vector (Amersham Pharmacia Biotech), and the resulting plasmid was transformed into *E. coli* strain BL21 (DE3) for overexpression of these proteins. Cells growing logarithmically were treated with 1 mM isopropyl-β-D-thiogalactopyranoside (IPTG) for 4 h at 30 °C. The fusion proteins were then purified by GST-tag affinity chromatography. Following purification of these antigens, immunization and sampling of the anti-sera from rabbit were performed by ABclonal, according to standard operating procedures. The specificity of the generated antibodies was determined by the manufacturer using ELISA and Western blotting.

### Western blotting

Equal amounts of proteins from H37Rv and H37Ra strains were separated on 10% or 15% SDS-PAGE gels. Proteins were stained with Coomassie Brilliant Blue R250 or transferred to polyvinylidene fluoride (PVDF) membranes (GE Healthcare). Membranes were blocked with 5% nonfat milk for 2 h, and incubated over-night with corresponding protein-specific antibodies (1:1000 dilution). After washing, membranes were incubated with a 1:3000 dilution of peroxidase-conjugated anti-rabbit IgG (Promega) for 1 h at room temperature. Chemiluminescence was detected by using the SuperSignal West Pico Chemiluminescent Substrate (Thermo Fisher Scientific), or alternatively visualized by using the DAB Horseradish Peroxidase Color Development Kit (Beyotime). Immunoblots were performed in three independent experiments, and bands of interest analyzed by ImageJ were expressed as mean ± SD.

### Northern blot hybridization

Total RNA (10 μg) from *M. tuberculosis* H37Rv or H37Ra and a low-range ssRNA Ladder (New England Biolabs) were denatured and separated on 8% polyacrylamide gels containing 8 M urea. The separated RNAs were electroblotted onto a Hybond-N+ membrane (Amersham) and cross-linked using short ultraviolet (UV) light. Specific DNA oligonucleotide probes for each candidate's sRNAs were radioactively labeled [γ-32P] ATP. Hybridization signals were exposed to a phosphor screen or determined by autoradiography on hyperfilms (Amersham). The hybridization signals on autoradiograms were scanned and digitized, and the density of each specific RNA band was quantified using ImageJ software. To account for loading variations, the signal intensity of each candidate sRNA was normalized to that of a reference RNA (5S rRNA) from the same lane.

### Rapid amplification of cDNA ends (RACE)

For 3′ RACE, a DNA adapter was ligated to total RNA from *M. tuberculosis* H37Rv or H37Ra, Reverse transcription was performed using 50 pmol of a single primer complementary to the DNA adapter. A no-reverse transcriptase control without Superscript III (Invitrogen) was included. PCR was performed using a forward primer specific to target sRNAs and reverse primer specific to the 3' RACE adapter. PCR products were separated on 8% DNA polyacrylamide gel and bands of interest were excised, cloned and sequenced.

### Construction of overexpression and knockdown strains

The vector was prepared as previously described by replacing the Hsp60 promoter in the pMV261 vector with the RrnB promoter (15). Using the primers listed in Table S1, the insert fragments of sRNA were generated by PCR and then cloned into the modified vector. The resultant transformants were selected on medium containing 20 μg/ml kanamycin. Finally, the correctly sequenced plasmid was transformed into *M. tuberculosis* H37Rv by electroporation.

### THP-1 monocyte cell infection

THP-1 cells were grown in RPMI 1640 supplemented with 10% heat-inactivated fetal bovine serum (Gibco). The THP-1 cells had been authenticated by short tandem repeat (STR) profiling and were confirmed to be free from *mycoplasma* contamination. THP-1 cells were inoculated in 24-well plates at 4 × 10^5^cells per well and differentiated in the presence of 0.1 μg/ml PMA (Phorbol-12-myristate-13-acetate) (Sigma) for 1 day. Following PBS washes, cells were infected with *M. tuberculosis* strains at a multiplicity of infection (MOI) of 1:10. After incubation at 37 °C for 4 h, extracellular bacteria were removed by washing with PBS, and THP-1 cells were incubated further. At designated time points, macrophages were lysed in 0.1% SDS, and lysates were plated on 7H10 agar for colony-forming unit (CFU) counts. Macrophage viability was assessed using the trypan blue dye exclusion method.

### Metabolic labeling and analysis of sulfolipid-1 (SL-1)

For the analysis of SL-1, *M. tuberculosis* cultures were metabolically labeled by supplementing the medium with [1-^14^C] propionic acid (5 Ci per 10 ml culture) during overnight incubation. Total lipids were subsequently extracted from the cell pellets with chloroform/methanol (2:1). The lipid extracts were concentrated, resolved by thin-layer chromatography (TLC) on silica gel plates using a chloroform/methanol/water (60:12:1) solvent system, and visualized by phosphorimaging to detect the radiolabeled SL-1.

### Statistical analysis

All experiments were independently at least three times (biological replicates), and data are presented as mean ± SD. Statistical analysis was performed using GraphPad Prism 8.0. Comparisons between two groups were conducted using unpaired two-tailed Student’s t-tests. For comparisons involving multiple groups, one-way ANOVA followed by Tukey's *post hoc* test was used. A value of *p*< 0.05 was considered statistically significant (∗*p*< 0.05, ∗∗*p*< 0.01, ∗∗∗*p*< 0.001).

### Data availability

RNA-seq data were deposited in the National Center for Biotechnology Information, under accession number PRJNA1196512 (https://www.ncbi.nlm.nih.gov/bioproject/?term=PRJNA1196512). The raw MS data have been deposited to the public access iprox database (http://www.iprox.org) with the identifier PXD064766.

## Supporting information

This article contains supporting information.

## Conflict of interest

The authors declare that they do not have any conflicts of interest with the content of this article.
