# Supplementary material for: Small RNA ASpks2 promotes Mycobacterium tuberculosis survival in macrophages via targeting polyketide synthase 2
Source: J Biol Chem. 2026 Apr 14;302(6):111449. doi: 10.1016/j.jbc.2026.111449 (PMC13194627; doi:10.1016/j.jbc.2026.111449)
Supplement: Figures S1–S5 [file mmc9.docx]

**Supplementary Figures**


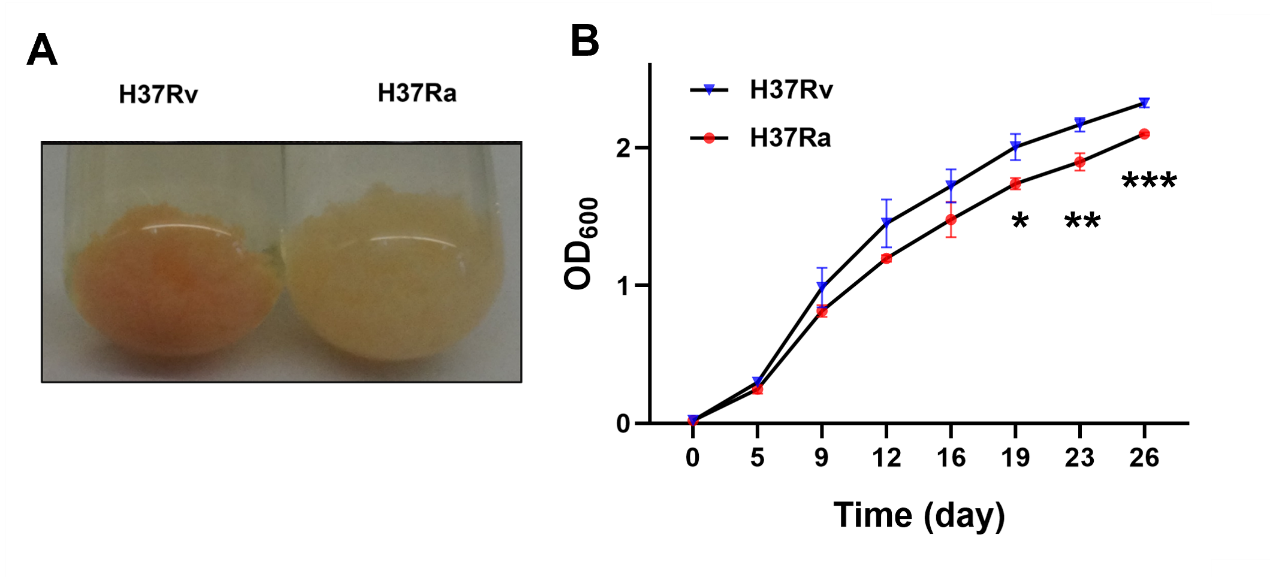


**Figure S1.** Phenotypic characterization of *M. tuberculosis* H37Rv and H37Ra strains. *A,* Cells were harvested and stained with 0.002% neutral red in barbital buffer. *B,* Growth curves of *M. tuberculosis* H37Rv and H37Ra in 7H9 medium (10% OADC + 0.5% glycerol, and 0.05% Tween 80). Statistical significance was determined using a two-tailed unpaired Student's t-test (* *p* < 0.05, ***p* < 0.01, ****p* < 0.001).

**
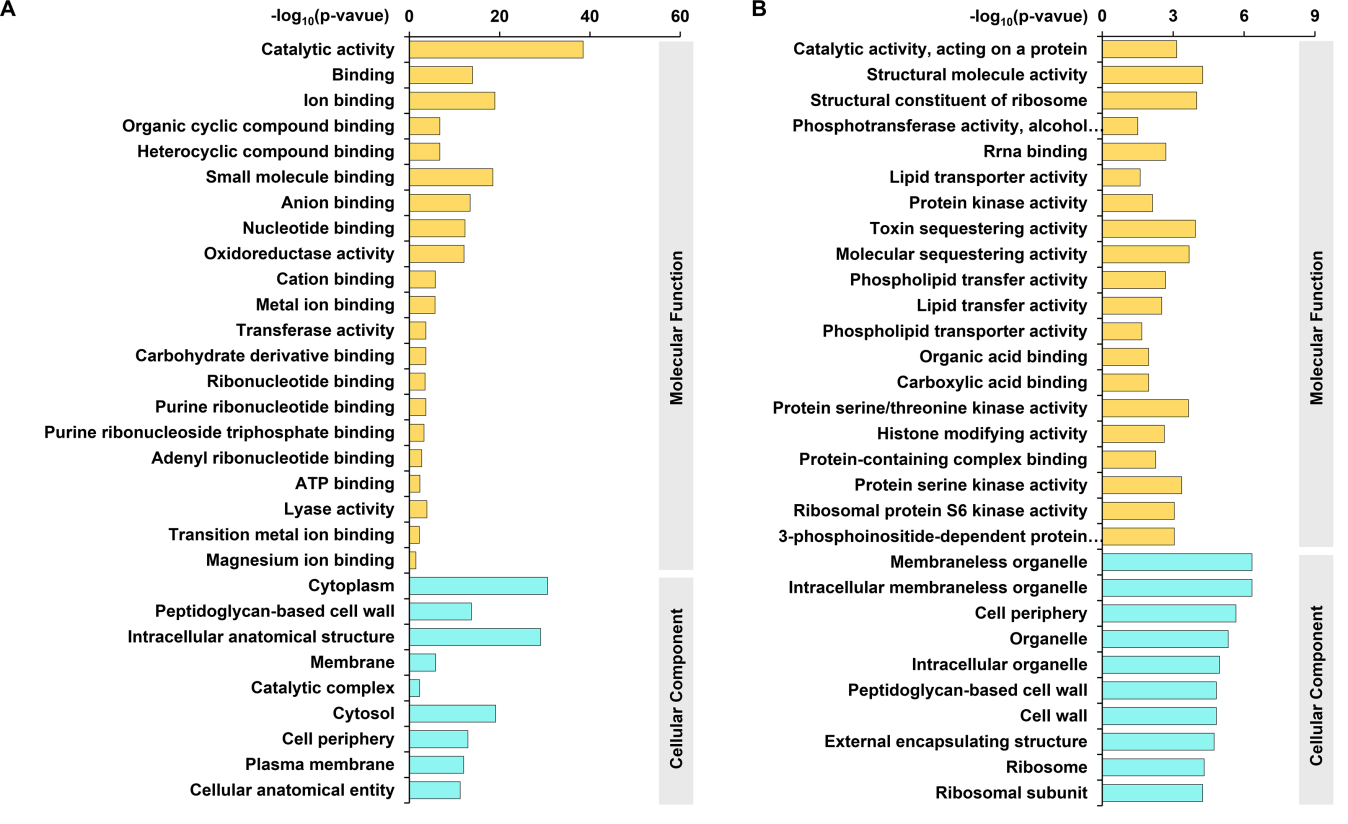
Figure S2.** GO enrichment analysis of differentially expressed proteins based on cellular components and molecular functions. *A,* up-regulated *B*, down-regulated.


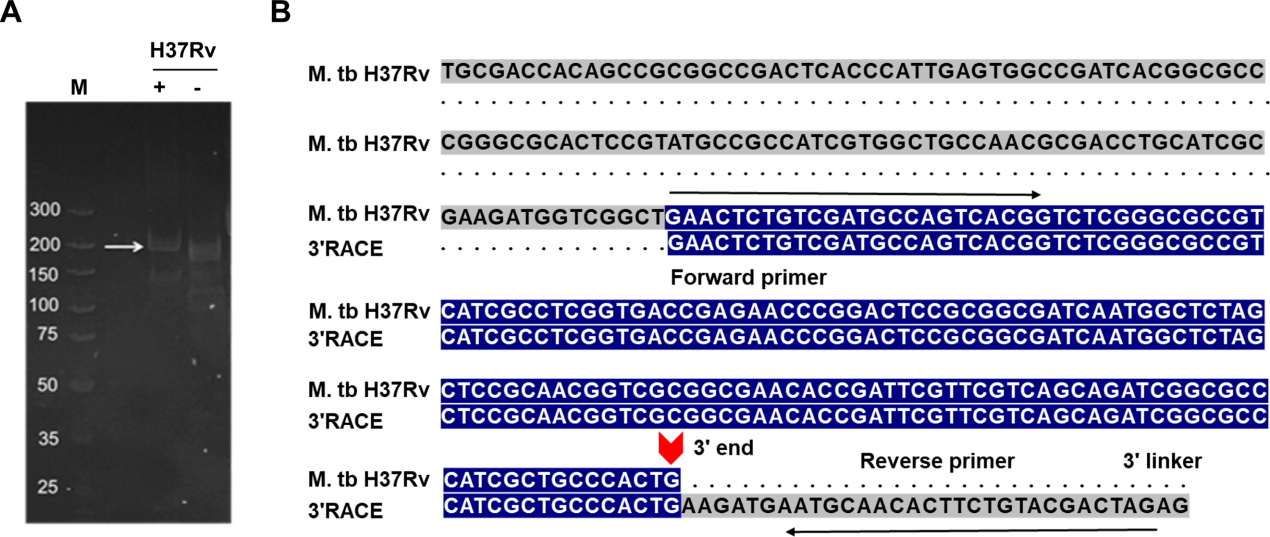


**Figure S3.** Verification of *ASpks2* by 3' RACE. *A*, 3ʹ RACE amplification of *ASpks2* 3' end. M: DNA molecular weight marker (bp); +, reverse transcription in the presence of reverse transcriptase; -, reverse transcription in the absence of reverse transcriptase. *B,* schematic representation of the *ASpks2* 3ʹ end.


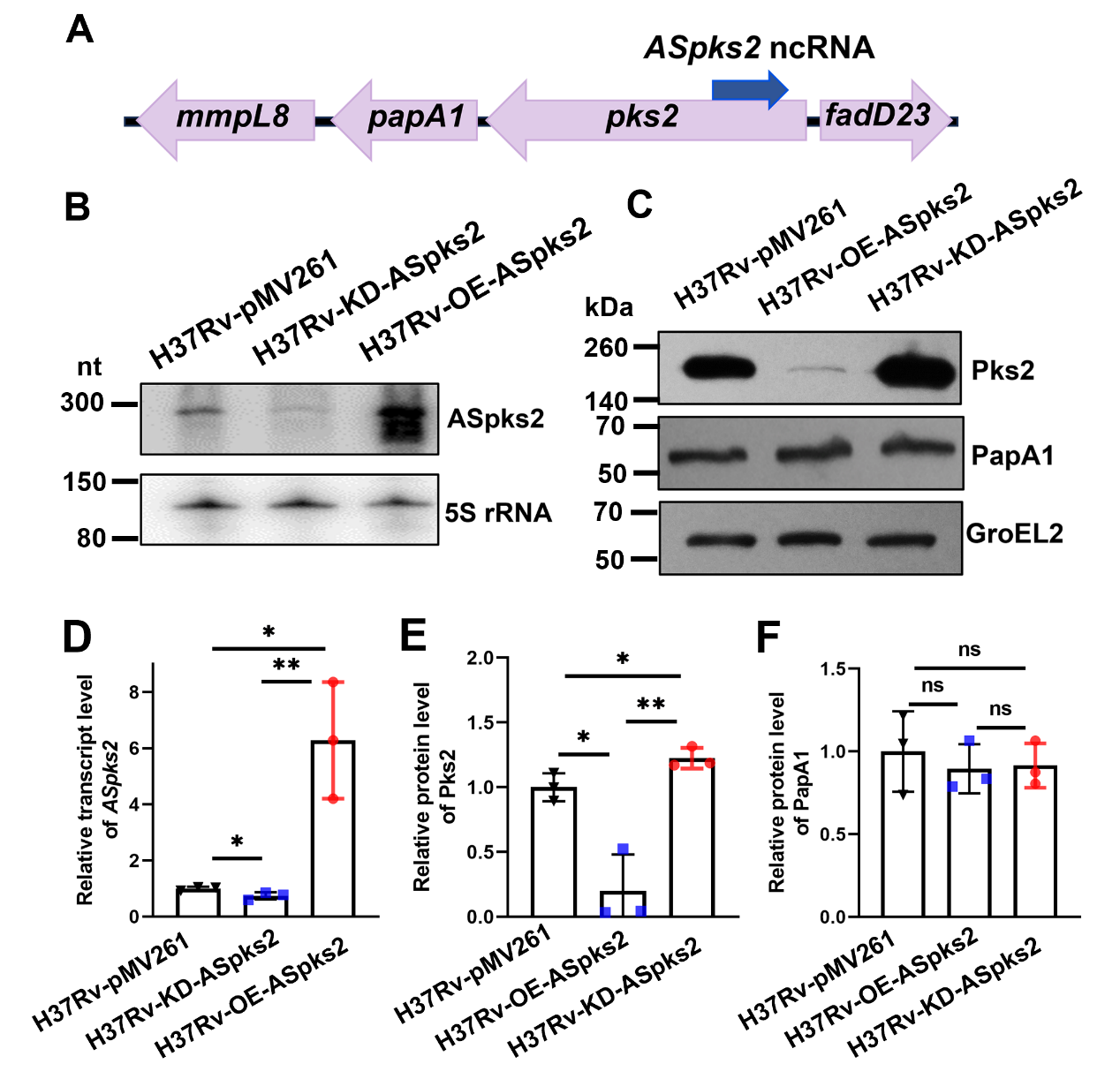


**Figure S4.** Molecular characterization of *ASpks2* expression in *M. tuberculosis* H37Rv strains. *A*, schematic representation of downstream or upstream of *pks2* locus. *B,* northern blot analysis of the *ASpks2* transcript in in the three strains (H37Rv-pMV261, H37Rv-KD-*ASpks2*, and H37Rv-OE-*ASpks2*). 5S rRNA was used as a loading control. *C*, western blotting of Pks2, PapA1 and GroEL2 in the three strains (H37Rv-pMV261, H37Rv-OE-*ASpks2*, and H37Rv-KD-*ASpks2*). *D*, Quantification of the Northern blot results shown in (B). The *ASpks2* transcript levels were quantified by densitometry, normalized to the 5S rRNA loading control, and then normalized to the expression level in the H37Rv-pMV261 strain. Statistical significance was determined using a two-tailed unpaired Student's t-test. *E* and *F*, Quantification of the Western blot results shown in (C). The protein levels of Pks2 (E) and PapA1 (F) were quantified by densitometry, normalized to the GroEL2 loading control, and then normalized to the protein level in the H37Rv-pMV261 strain. Data are presented as mean ± SD from three independent experiments. Statistical significance was determined by One-way analysis of variance (ANOVA) followed by Tukey's test (ns: no significance, * *p* < 0.05, ***p* < 0.01).


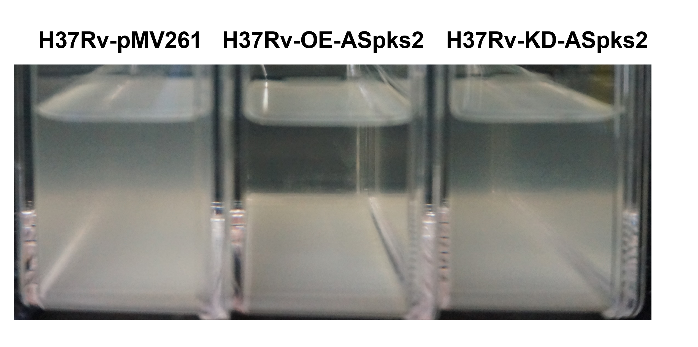


**Figure S5**. Analysis of bacterial aggregation in *M. tuberculosis* H37Rv strains. The indicated strains (H37Rv-pMV261, H37Rv-OE-*ASpks2*, and H37Rv-KD-*ASpks2*) were cultured statically in 7H9 liquid medium (10% OADC + 0.5% glycerol, and 0.05% Tween 80) for 4 weeks. Bacterial aggregation phenotypes were then observed and documented.
